# Supplementary material for: Are carbon emissions trading and green financial instruments synergistic? -Comprehensive quantitative research based on content analysis
Source: PLoS One. 2024 Mar 7;19(3):e0298601. doi: 10.1371/journal.pone.0298601 (PMC10919601; doi:10.1371/journal.pone.0298601)
Supplement: S1 Data — (ZIP) [file pone.0298601.s001.zip › Data/地级市Z_F工作报告.zip!/░▓╗╒/┘±╓▌╩╨/└┤╫╘╣1⁄2╓┌║┼í╛┬φ┐╦╩2╛▌═°í┐_2008.docx]

各位代表：
　　现在，我代表市人民政府，向大会报告工作，请予审议，并请列席人员提出意见。
　　一、今年以来的工作回顾
　　今年以来，在市委的坚强领导下，市政府紧紧围绕市二届人大三次会议确定的各项工作任务，团结和带领全市人民，坚持以科学发展观为指导，求真务实，顽强拼搏，经济社会发展呈现速度加快、质量提高、后劲增强、民生改善的良好态势。初步核算，上半年完成生产总值194.78亿元，同比增长11.3%。城镇居民人均可支配收入5785元，增长14%；农民人均现金收入2264元，增长24%；实现进出口总额4849万美元，增长49.4％；其中出口3710万美元，增长58.2%，高于全省平均水平19.8个百分点。1-8月份，完成全部财政收入16.94亿元，增长30.1%；其中地方财政收入8.98亿元，增长30.7%。
　　（一）结构调整步伐加快，运行质量明显提高
　　工业经济提速增效。1-8月份，全市累计实现规模以上工业增加值36.45亿元，增长28.5%；新增规模以上工业企业34家。50家“125”行动计划重点培育企业完成工业总产值66.71亿元，增长19.8%。上半年，全市工业化率达20.6 %，同比提高1.4个百分点。规模以上工业企业实现利润2.97亿元，增长2.5倍。工业经济效益综合指数达177.23%，同比提高42.5个百分点，为近年来最好水平。
　　“三农”工作再创佳绩。夏粮连续5年增产增收，总产283.1万吨，平均亩产448公斤，再创历史新高；其中小麦平均亩产452公斤，居全省第一位。畜牧生产全面回升，上半年，肉、蛋、奶产量分别增长10.6％、8.6％和7.0％；新增规模养殖场（户）340家。新增农民专业合作组织92家、国家级农业产业化龙头企业1家、省级龙头企业7家，市级以上农业产业化龙头企业销售收入同比增长25％以上。涡阳高炉镇陆杨村、蒙城小涧镇尖山村等4个村入选省级特色产业示范村，利辛王人镇吴营村、谯城区城父镇刘楼村等成为全市社会主义新农村建设新典型。全省新农村建设暨为民服务全程代理工作现场会和新型农村合作医疗工作会议在我市召开，谯城区为民服务全程代理和新农合工作经验在全省推广。
　　第三产业繁荣活跃。1-8月份，全市实现社会消费品零售总额102.06亿元，增长22.1%。新增“万村千乡试点工程”连锁农家店310家，专业市场建设和现代物流业取得新进展。上半年，实现国内旅游收入5.5亿元，增长33.9%。农发行亳州市分行、邮储银行亳州市分行正式成立，建行亳州市分行、徽商银行亳州市支行、华安证券亳州营业部获准设立，国元保险入驻亳州，市信用担保投资公司挂牌成立；全市金融机构各项存款、贷款余额和各类保费收入分别比上年同期增加50.7亿元、17.7亿元和4亿元。交通运输、邮电通信、住宿餐饮、房地产等服务业保持了较快增长势头。
　　（二）城市功能日益完善，发展载体不断夯实
　　城市建设亮点纷呈。全市新建、续建城市基础设施重点项目56个，完成投资7.1亿元。人民群众多年期盼的魏武广场建成开放，宋汤河城区段改造二期工程基本竣工；涡河城区段治理拆迁工作顺利完成，居民还原房工程如期开工；北关历史街区修复工作进度加快，芍花西路建设、人民路西段和汤王大道配套完善工程顺利实施；市南部新区控制性规划加紧编制，希夷大道、魏武大道向南延伸工程即将开工；市区及三县城市园林绿化、道路硬化亮化、供水及污水管网改造等工程加快实施。土地利用总体规划修编工作稳步推进，涡阳县、蒙城县被列为省规划修编试点县。城市综合执法、土地管理和环境整治力度不断加大，打击土地非法买卖、非法占用专项行动深入实施，文明创建工作取得了积极进展。
　　园区建设扎实推进。市经济开发区路网设计及控制性详规调整工作全面完成，药都大道东段工程、亳涡公路开发区段改造、还原小区建设等重点项目前期工作顺利实施。亳州工业园完成基础设施投资3.2亿元，建成区面积超过3平方公里；蒙城经济开发区和利辛工业园区分别完成投资3.9亿元和1.4亿元，建成区面积均超过2平方公里；涡阳工业园迁址获省政府批准，机械加工等工业小区建设步伐加快。1-8月份，县区工业园区共新建道路27公里、标准厂房4.8万平方米，新批准进园企业36家，新开工项目38个，新投产及试产企业28家。
　　（三）对外开放不断扩大，经济发展后劲增强
　　招商引资成效明显。1-8月份，落实招商引资项目348个，到位资金62.6亿元；实际利用外资5615万美元，增长45.3 %。成功举办了2008年国际（亳州）中医药博览会暨第24届全国（亳州）中药材交易会，共签约项目64个，比上年增加37个；总投资44.2亿元，是上年的2.47倍；其中，合同签约项目54个，比上年增加45个；合同投资32.9亿元，是上年的5.23倍。签约项目中，总投资5000万元以上项目26个、亿元以上项目10个。本届药博会参会客商之众、签约项目之多、项目档次之高、引资金额之大，均创历史新高，进一步彰显出亳州作为“中华药都”的风采和魅力。
　　项目建设力度加大。1-8月份，完成固定资产投资61.21亿元，增长34.3%。列入省“861”行动计划的18个项目，累计完成投资18亿元，增长69.1%；列入市“1468”战略布局的93个项目，累计完成投资28.5亿元，增长17.2%。江苏雨润集团在蒙城投资3亿元、年产3万吨休闲食品项目顺利开工；亳州现代中药产业基地、三星化工“586”项目、板集电厂等一批重点项目扎实推进；板集煤矿、刘店煤矿、金源化工二甲醚项目、东升肉鸡深加工项目等正在加紧建设，明年上半年可望投产；投资亿元以上的中能安驰汽车制造、森威板业、全森木业等项目基本建成，成为经济发展中的新亮点。
　　（四）人民生活不断改善，社会事业全面发展
　　民生工程取得新进展。1-8月份，共发放农村低保金5353万元、五保供养资金2351万元，保障了18.6万农村低保对象和3.2万名农村“五保户”的基本生活；实现了新农合县区全覆盖，参合农民435万人，累计支付补偿资金1.67亿元，受益群众23.4万人次；新改造项目学校75个，提前6个月全面消除农村中小学D级危房；拨付城乡义务教育保障经费1.8亿元，受益学生75.4万人；资助高校、中职学校家庭困难学生8.2万人次，发放资助金1251万元；开工建设廉租住房438套，发放廉租房补贴105万元；开工建设农村饮水安全工程项目64个，完成投资5289万元；建成农村公路“村村通”工程668.6公里，完成投资2.3亿元；改造市区自来水支管网124公里，完成投资2389万元；“十八项民生工程”和市政府承诺的“十件实事”其他项目也都按序时进度积极推进。就业和社会保障工作取得新成绩。1-8月份，全市城镇新增就业1.87万人，实现下岗失业人员再就业5313人，新增转移农村劳动力5.8万人，发放小额担保贷款3389万元；发放养老保险金2.2亿元、失业保险金427万元，落实农民工工资保障金227万元；新增城镇居民医疗保险参保人数6.1万，参保总数达23.8万人。
　　社会事业实现新发展。坚持全民发动、群防群治，战胜了突如其来的手足口病疫情；扎实推进乡镇卫生院、村卫生室和标准化社区卫生服务中心（站）建设，城乡公共卫生服务体系进一步完善。继续加大对教育的投入，69个寄宿制项目学校、1910个农村现代远程教育项目学校全部建成，13个改善城区义务教育办学条件项目中有10个基本完工，亳州师专新校区建设前期工作加紧推进；高考本科录取人数达8913人，创建市以来最好水平。科技工作不断加强，11个项目列入国家和省科技计划。群众性文化活动广泛开展，“二夹弦”被列为第二批国家级非物质文化遗产。迎奥运全民健身活动深入开展,竞技体育成绩显著。计划生育工作扎实有效，出生人口性别比稳中有降，低生育水平保持稳定。环保工作力度加大，生态市建设步伐加快。国防后备力量建设进一步加强，人民防空、民兵预备役和拥军优属工作取得新成绩。妇女儿童、老龄、残疾人等事业取得新进展，人事、编制、工商、税务、财政、审计、物价、统计、质监、外事、侨务、对台、档案、地方志、无线电管理和机关事务管理等各项工作取得新成效。
　　社会管理水平得到新提高。大力推进依法治市，深入开展反腐倡廉制度建设推进年活动，政府自身建设得到加强，服务水平和行政效率不断提高。自觉接受人大、政协监督，办理人大代表议案、建议116件和政协委员提案288件。扎实开展安全生产“隐患治理年”活动，各类安全生产事故起数和死亡人数同比分别下降52.2%和32.8%。继续推进公安“三基”工程建设，强化社会治安综合治理，加强信访维稳工作和宗教事务管理，完善应急管理机制，社会秩序保持稳定，人民群众的安全感进一步提高。
　　各位代表，亳州的发展，是省委、省政府和市委正确领导的结果，是市人大、市政协监督支持和社会各界关心帮助的结果，是历届老领导和老同志支持、帮助的结果，是全市人民奋发努力、团结拼搏的结果。在此,我代表市人民政府，向全市广大干部群众，向各民主党派、工商联和人民团体，向驻亳解放军、武警官兵和公安政法干警，向所有参与、支持、帮助亳州发展的各界朋友,致以崇高的敬意和衷心的感谢!
　　回顾今年以来的工作，我们也清醒地认识到我市经济社会发展中还存在一些矛盾和问题，主要表现在：经济结构不尽合理，工业总量偏小，整体实力不强；土地、资金、环保等瓶颈制约还未得到根本缓解，招商引资和项目建设有待进一步加强；社会事业和公共基础设施欠账较多，改善民生的任务还很艰巨，影响和谐稳定的因素还不同程度地存在；思想解放的深度和改革开放的力度还不够，机关作风和效能建设有待加强，发展环境亟须进一步优化。对此，我们将高度重视，采取有效措施，认真加以解决，不辜负全市人民的期望！
　　二、确保完成全年各项目标任务
　　各位代表，我市正处在科学发展、和谐发展、跨越发展的重要时期，既面临着广阔的空间和难得的机遇，又面临着各种困难和挑战。面对复杂多样的国内外环境，面对周边地市竞相发展的竞争态势，不进则退，慢进也是退。我们必须认真落实胡锦涛总书记视察安徽和王金山书记考察亳州时的重要讲话精神，进一步增强大局意识、忧患意识和责任意识，紧紧抓住中部崛起和省委、省政府重点支持皖北三市发展的政策机遇，按照市委提出的“发展为上、民生为先、稳定为重、少说多做、真抓实干”的总体部署，解放思想、开拓进取，卧薪尝胆、奋发有为，努力缩小发展差距，赶上全省发展步伐。
　　当前，最为紧要的是，全市上下要按照年初市政府工作报告和全市第二季度经济工作督查调度会的要求，扎扎实实做好年内各项工作，确保完成全年各项目标任务。
　　（一）突出工业强市，加快新型工业化进程
　　我市经济发展的差距主要在工业，加快发展的潜力和希望也在工业。我们要把工业强市作为发展的主导战略和主攻方向，把招商引资和项目建设作为工业发展的主要抓手，把工业园区作为聚集资源、引领发展的重要引擎，坚持主抓工业不动摇，努力走出一条符合亳州实际的新型工业化道路。
　　加快提升“125”行动计划。围绕“1468”战略布局和“一都一城一基地”建设，加强对50家“125”行动计划重点培育企业的指导、协调和服务，把骨干企业做大做强，把中小企业做专做精，把特色企业做优做多，力争全年新增规模工业企业50家以上、规模工业增加值增长30％以上。要进一步做好工业生产大宗原材料及煤、电、油、运等调度工作，改善服务，排忧解难，引导企业开拓市场、扩大经营、提高效益。
　　狠抓重点项目不放松。继续实行重大项目包保责任制，完善项目工作协调调度机制，突出抓好列入省“861”行动计划和市“1468”战略布局的重点项目，全力推进亳州现代中药产业基地、刘店煤矿、板集煤矿、中能安驰汽车生产、瑞福祥小麦深加工二期工程等项目建设，重点抓好2008年徽商大会、厦洽会和药博会签约项目落地，确保全年完成固定资产投资115亿元以上。加快推进三星化工与河南永煤集团战略性合作取得实质性进展，争取超百亿元煤化工项目尽快开工。着力推动与淮北矿业集团的务实合作，完善利益分配机制，增强煤炭资源开发对地方经济发展的贡献能力。抓住省重点支持皖北三市发展的机遇，积极主动搞好对接，争取上马一批新建项目，储备一批待建项目，以项目拉动投资，以投资促进发展。
　　着力壮大县域经济。坚持把工业园区和产业发展结合起来，加强规划引导，完善配套设施，落实优惠政策，优化发展环境，促进项目向园区集中、园区向专业化发展、产业向上下游延伸，重点培育壮大谯城区现代中药、蒙城县汽车制造改装、涡阳县煤化工、利辛丝网纱门等一批各具特色的产业园区，力争县域规模以上工业增长30％以上。
　　推进科技创新和节能减排。建立科技创新基金，引导和支持创新要素向企业聚集，促进科技成果向现实生产力转化。积极争取成立省级中药研究所，加快建设一批国家级、省级技术检测和产品研发中心，推进市级科研孵化器建设，加强与中国中医科学院、南京中医药大学、安徽中医学院、合肥工业大学等科研院所合作，促进产学研合作向广度深度发展。落实循环经济促进法，加快古井热电公司、三星化工集团节能技改工程和利辛县污水处理厂建设，确保实现年度节能减排目标。
　　（二）积极发展现代农业，推进社会主义新农村建设
　　“三农”工作事关全市改革发展稳定大局。我们要始终坚持以社会主义新农村建设为主线，以农民增收为核心，以农业产业化为抓手，促进农业发展更快、农民增收更多、农村更加繁荣稳定。
　　大力发展现代农业。积极调整秋季作物种植结构，在抓好优质粮食生产的同时，进一步做大中药材、蔬菜、林木、畜牧等特色产业，推进农业集约化、产业化、标准化、品牌化生产。继续推行“一村一品”，重点支持省市级102个试点村和12个示范镇的产业培育，力争年内形成专业村镇300个。实施农业产业化提升工程，通过外引内联，着力培育壮大一批粮食、林木、中药材、肉食品加工龙头企业，力争全年实现农产品加工产值100亿元以上。积极发展农民专业合作组织，稳步推进土地适度规模经营。加强以农田水利为重点的农业基础建设，继续推进低洼地治理，搞好大中沟疏浚配套，大力推进农田林网建设，确保年内新增除涝面积21万亩、新建和完善农田林网27万亩。大力实施“阳光工程”和新型农民技能培训，提高农村劳动力转移的组织化程度。
　　积极推进社会主义新农村建设。加快村镇规划编制步伐，大力开展空心村整治，实施土地开发整理，用足用活城乡建设用地增减挂钩试点、土地复垦置换等政策，统筹城乡建设。加强农村道路、饮水、通讯、电力等基础设施建设，加快发展农村教育、文化、卫生等各项社会事业，推进城乡一体化进程。深入实施乡村清洁工程，扎实开展农村环保小康行动，不断改善农村生产生活环境。完善为民服务全程代理制，深化农村综合改革，加快建立农村工作新机制。进一步完善多元投资、社会共建的新农村建设推进机制，抓好企业帮扶、单位帮建、项目捆绑、资金整合等措施落实，让更多群众享受新农村建设的成果。广大农村基层干部是新农村建设的骨干力量，工作任务十分繁重，各级政府要真心真意地关心他们的生活，满腔热情地支持他们开展工作。
　　（三）突出发展现代服务业，提升第三产业水平
　　当前，我市正处于工业化、城镇化加速发展时期。我们要继续坚持以市场化、产业化、社会化为方向，大力发展面向生产、面向民生的现代服务业，不断培育新的增长点。
　　加快发展文化旅游业。加快以华佗中医药文化博物馆、曹操纪念馆、涡阳老子文化园、蒙城庄子祠、利辛西淝河生态旅游为重点的文化旅游项目建设，建成一批新的旅游景点。大力开展宣传促销，加强区域交流合作。积极筹建亳州文化旅游投资公司，整合现有文化、旅游资源，通过市场化运作，精心打造一批文化旅游品牌，努力实现旅游业大发展、大提升。
　　大力发展金融、证券和保险业。抓好徽商银行、华安证券入驻及工行亳州支行升格工作，组建谯城区农村合作银行，完成涡阳县、利辛县农村信用社票据兑付工作。引导各金融机构不断加大对“三农”和中小企业的支持力度，确保完成已安排的全年新增贷款40亿元任务。支持国元保险等保险业机构发展“三农”保险，开发符合中小企业需要的保险品种。加强银政企合作，整合政府融资平台，完善信用担保体系，营造良好的金融生态环境。
　　改造提升商贸物流业。继续实施“万村千乡市场工程”，加强各类市场建设，健全城乡生活和生产资料营销网络，确保年内建设或改造农家店500家以上。加快电子支付平台建设，实施药材质量信息化管理，提高中国（亳州）中药材交易中心现代化服务水平。积极引进家乐福、苏果等大型商贸流通企业，整合、改造现有物流企业，加速发展现代物流业。
　　（四）加强城市建设与管理，提高综合承载能力
　　城市是经济社会发展的重要载体。我们要继续坚持走城市化带动发展的路子，以城市大建设促进城市规模大扩展、功能大完善、品位大提升、经济大发展。
　　着力加大城市建设力度。年内开工魏武大道、希夷大道向南延伸工程，启动105国道与307省道连接线工程，加快市南部新区建设，增强全市人民的发展信心。坚持开发与保护并重，推进亳州北关历史街区修复工程建设，继续搞好老城区硬化、美化、绿化和亮化工作。加快涡河城区段治理、城区道路配套完善和污水管网等工程建设，确保完成年度目标任务。加快供水管网建设，积极筹建涡北水厂，力争尽快实现市区公共供水全覆盖。年内开工亳州火车站改扩建项目，争取许泗高速亳州段批准筹建。加快三县县城建设步伐，完善功能，做大规模，聚集人气，构筑县域经济发展的坚实平台。
　　努力提升城市管理水平。加强城市管理综合执法，理顺关系，提高效率，建立市区联动、调控有力的长效机制。强化土地管理，盘活闲置土地，扎实开展打击土地“两非”专项行动，下决心净化土地市场。继续开展“扮靓城市，爱我家园”活动，下大气力整治市容市貌、环境卫生、交通秩序和市场秩序，确保完成争创全省文明城市年度目标任务。
　　（五）深入推进改革开放，增强经济发展活力
　　加快亳州发展，必须坚定不移地深化改革、扩大开放，敢于用改革创新的思维解决发展中的问题，善于借开放搞活的力量弥补发展中的不足。只有这样，亳州经济社会发展才能焕发出勃勃生机和持久活力！
　　加快以企业为重点的改革攻坚步伐。按照“彻底改、改彻底”和“三个置换、两个结合、一个体系建设”（即：政府置换土地、企业置换产权、职工置换身份，企业改革与招商引资相结合、与经济结构战略性调整相结合，建立健全社会保障体系）的工作要求，加快古井集团产权改革、瑞福祥公司资产置换、九方制药公司资产重组等改制工作，集中力量，合力攻坚，尽快完成市及县、区属国有集体企业改革任务。加快公共服务市场化进程，推进城市供水、供气、供热、公交、污水和垃圾处理等方面的改革。
　　不断提高对外开放水平。坚持“全面发动、全员参与、完善机制、优化环境”的工作思路，采取以商招商、上门招商等形式，突出产业招商、节会招商和亲情招商，招老乡，招大商，进一步掀起招商引资新高潮，争取在大项目引进上取得实质性进展，力争全年招商引资突破100亿元。加快转变外贸增长方式，优化出口商品结构，稳步扩大中药材、农副产品等特色产品出口，确保全年实现进出口总额9000万美元以上。
　　深入推进全民创业。坚持全民创业、企业为本，实施中小企业成长工程，建设各类创业基地，为全民创业搭建良好平台。借鉴农村为民服务全程代理工作经验，实施创业全程代理服务。大力发展非公有制经济，落实促进政策，强化引导扶持，优化创业环境，鼓励冒尖，宽容失败，进一步营造创新可贵、创业光荣、尊重企业家的社会氛围，让企业家放手放胆放心发展。
　　（六）扎实推进民生工程，维护广大群众切身利益
　　今年实施的“十八项民生工程”和“十件实事”，牵涉面广、投入量大、工作任务艰巨。但是，无论这些工作有多复杂、多艰难，我们都必须服从全省、全市工作大局，带着责任、带着感情、带着智慧、带着精神，全力以赴抓好各项民生工程，真正把实事办好、好事办实，努力让全市人民共享亳州改革发展的成果。
　　全力推进“十八项民生工程”和“十件实事”。拓宽筹资渠道，严格建设标准，强化质量监管，确保完成全年1000公里农村公路“村村通”工程建设任务，解决22万人农村人口饮水安全问题，实现新建或改扩建乡镇卫生院20所、村卫生室228个、社区卫生服务机构18个的年度目标。进一步提高农村五保户供养标准和集中供养率，建成二级以上标准化乡镇敬老院30所，年内实现每个乡镇至少有1所二级以上标准化敬老院的工作目标。落实农村计划生育家庭奖扶政策，确保全市4415名奖扶对象资金的足额及时发放。全面实施城镇居民基本医疗保险制度，确保年内覆盖率达90%。全面开工建设廉租房，确保完成1700户城市低保住房困难家庭住房补贴发放任务。总之，要进一步明确目标责任，加快工作进度，千方百计地完成“十八项民生工程”和“十件实事”年度目标任务。
　　加强就业和社会保障工作。坚持以创业促就业、以发展带就业，强化就业和再就业培训，完善困难群众就业援助制度，重点促进“零就业”家庭就业，确保年内城镇新增就业2万人以上、城镇登记失业率控制在4％以内。加快建立覆盖城乡的社会保障体系，扩大养老、医疗、失业、工伤、生育等保险范围。高度关注社会弱势群体，落实被征地农民社会保障办法，健全城乡居民最低生活保障制度，不断提高城乡医疗救助水平。
　　（七）全面发展各项社会事业，推进和谐亳州建设
　　加快发展社会事业是统筹经济社会发展、统筹城乡发展的重要内容。我们要不断加大社会事业投入，切实加强社会管理，努力为全市人民提供更多、更优质的公共产品和社会服务，促进经济社会全面协调可持续发展。
　　加快发展各项社会事业。坚持教育优先发展，加大教育投入，继续改善农村中小学办学条件，扩大城区公办义务教育，推进义务教育均衡发展；加快发展高中阶段教育，大力发展职业技术教育；积极发展高等教育，促进亳州师专新校区建设年内取得实质性进展。大力发展卫生事业，鼓励社会力量创办民营或股份制医院，积极筹建市人民医院新院区，强化重大疾病预防和控制，努力提高公共卫生服务能力；推广谯城区经验，提升新农合工作水平。积极开展群众性文化活动，突出抓好社区和乡镇综合文化站、文化信息资源共享、农村电影放映和送书下乡等工程，加快市博物馆、图书馆筹建步伐。广泛开展以华佗五禽戏为代表的全民健身活动，提高竞技体育水平。加强计划生育基层基础工作，强化流动人口管理，降低出生人口性别比，努力提高计划生育管理水平。依法维护妇女儿童合法权益，高度关注农村留守儿童的教育和成长，积极发展残疾人和老龄事业，支持社会力量兴办福利和慈善事业。加强国防教育和后备力量建设，做好人民防空、民兵预备役和拥军优属工作。加强统计工作，精心组织好第二次全国经济普查。进一步做好人事、编制、工商、税务、财政、审计、物价、气象、农机、质监、外事、侨务、对台、档案、地方志、扶贫开发、无线电管理和机关事务管理等各项工作。
　　切实加强社会管理。坚持依法治市，大力开展“五五”普法活动，努力提高全社会法治意识。强化突发公共事件应急管理，增强维护公共安全与社会稳定的能力。严格信访工作目标责任管理，完善领导开门接访、带案下访和包案处理制度，努力实现信访“降量退位”。全面完成公安“三基”工程建设目标任务，继续加强社会治安综合治理，严厉打击各类刑事犯罪，依法管理宗教事务，努力建设“平安亳州”。落实安全生产责任制和责任追究制，抓好重点区域、重点行业的隐患排查与治理，确保每亿元GDP生产安全事故死亡率控制在0.8以内。强化市场监管，依法打击涉众型经济犯罪、制假售假、偷逃骗税等违法活动，重点整顿食品药品市场，规范市场经济秩序，营造良好的市场环境。
　　三、努力建设人民满意的政府
　　我们的政府是人民的政府，对人民负责、让人民满意是我们的神圣职责。面对跨越发展的新任务，面对激烈竞争的新态势，面对人民群众的新期待，我们必须切实加强政府自身建设，按照有作为、高效率、作风实、从政廉的要求，全面提高施政水平。
　　（一）解放思想，奋发有为
　　思想解放的程度，决定经济社会发展的速度。要改变亳州落后面貌，我们必须坚持把解放思想、奋发有为贯穿于政府工作的始终，用创新的思维拓展思路，用创新的制度激发活力，用创新的办法破解难题。以时不我待、自我加压、奋起直追的勇气，以无私无畏、勇往直前、坚定改革的锐气，以雷厉风行、真抓实干、一抓到底的风气，创造出无愧于人民、无愧于历史的新业绩。要始终保持昂扬向上、迎难而上的精神状态，把发展作为第一要务，把招商作为第一要事，树立大投资、大招商、大建设的发展意识，突出一个“敢”字，破除一个“难”字，落实一个“干”字，努力形成只争朝夕、攻坚克难、争先进位的工作局面。唯有如此，才能有所创新、有所突破；唯有如此，亳州的发展才大有可为、大有希望！
　　（二）致力发展，科学发展
　　当前，亳州最大的问题是发展不快、发展不优的问题，最为紧迫的任务是加快发展、科学发展。我们必须把加快发展作为第一追求，把服务发展作为第一任务，聚精会神搞建设，一心一意谋发展。全市各级政府既要着眼长远，谋划好牵动全局、关系亳州未来发展的大事；又要立足当前，抓好近两三年支撑亳州发展的一批大项目、大产业。要加快煤炭资源开发，尽快形成超千万吨煤炭生产能力；加快涡阳煤炭综合利用循环经济产业园建设，争取亳州板集电厂、涡阳三星百亿元煤化工基地尽快开工，把煤电、煤化工打造成亳州第一支柱产业。打好“中华药都”这一品牌，引进国内知名制药企业，发展中药材精深加工，建设国家现代中药产业基地，形成独具特色的支柱产业。进一步扩大古井集团、双轮集团等企业的市场份额和品牌影响力，重振白酒产业辉煌；加快农副产品深加工和资源转化，做大做强一批粮食、肉食、林木、蔬菜加工等农业产业化龙头企业，形成市场竞争力较强的传统支柱产业。抓好汽车制造、中重型汽车改装和零部件加工等重点项目，把汽车产业打造成为亳州新兴的支柱产业。坚持名城保护、新区开发、老城改造相结合，突出抓好南部新区建设，完善城市基础设施，加快市博物馆、图书馆、体育馆、人民医院新院区等项目建设，大力发展文化、旅游、商贸、物流等产业，努力把亳州建设成为皖西北新兴的工业和商贸城市，再现亳州这座国家级历史文化名城的繁荣与魅力。总之，通过加快工业化、城镇化和农业产业化发展步伐，努力实现三年内全市工业增加值和固定资产投资在2007年基础上翻一番、城市面貌大变样的目标，不断开创亳州加快发展的新局面。
　　（三）改进作风，务实高效
　　优良的工作作风是凝聚民心的巨大力量。我们要继续坚持说实话、办实事、求实效，坚决反对搞形式主义、摆花架子、做表面文章，推广“一线工作法”，努力做到问题在一线解决、业绩在一线创造。进一步加强行政服务中心建设，减少审批事项，规范行政行为，提高工作效率。加强机关效能建设，健全绩效考核体系，加大政务督查力度，强化工作执行力。进一步扩大政务公开范围，办好政府网站和行风热线，整合政务热线平台，提高政府工作的透明度，确保权力在阳光下运行。加强政府诚信建设，政府承诺的每件事情都要有诺必践、有践必果，对一切破坏发展环境的行为都要坚决惩处、决不姑息，以敢于负责的精神和实实在在的政绩取信于民、造福于民。
　　（四）依法行政，廉洁从政
　　 严格按照法定权限和程序行使权力、履行职责，自觉贯彻落实市委的各项决策和部署，自觉接受人大的法律监督和工作监督、政协的民主监督以及社会各界的监督，认真办理人大代表议案建议和政协委员提案，广泛听取各民主党派、工商联和无党派人士的意见和建议，充分发挥行政监察和审计监督的作用。继续完善重大事项集体决策、专家论证、社会公示和决策评估机制，不断提高科学决策、民主决策水平。深入开展反腐倡廉制度建设推进年活动，切实做到用制度管权、按制度办事、靠制度管人。严格执行廉洁自律各项规定，坚决抵制和反对一切消极腐败现象，坚决纠正各种损害群众利益的不正之风，坚决查处各类违法违纪案件。积极建设节约型政府，坚持勤俭办一切事情，把有限的资金用在加快发展、改善民生上。全面加强政府工作人员教育、培训和管理，提高公务员素质，努力建设一支勤政为民、务实清廉的公务员队伍。
　　各位代表，当前亳州经济社会发展已经进入一个新的阶段，各级政府肩负的责任更大、任务更重。让我们在市委的坚强领导下，以党的十七大和胡锦涛总书记视察安徽时的重要讲话精神为指导，深入贯彻落实科学发展观，解放思想，奋发有为，真抓实干，锐意进取，为建设富强、民主、文明、和谐的新亳州而努力奋斗！
